# Acute HIV infection presenting as hemophagocytic syndrome with an unusual serological and virological response to ART

**DOI:** 10.1186/s12879-016-1945-9

**Published:** 2016-10-28

**Authors:** Rita Veiga Ferraz, Ana Cláudia Carvalho, Fernando Araújo, Carmo Koch, Cândida Abreu, António Sarmento

**Affiliations:** 1 Infectious Diseases Department, Centro Hospitalar de São João, Alameda Prof. Hernâni Monteiro, Porto, Portugal; 2 Instituto de Inovação e Investigação em Saúde (I3S). Grupo de I&D em Nefrologia e Doenças Infeciosas. Instituto Nacional de Engenharia Biomédica (INEB), Porto, Portugal; 3 Transfusion Medicine and Hematology Department, Centro Hospitalar de São João, Porto, Portugal

**Keywords:** HIV, Acute infection, Haemophagocytic syndrome

## Abstract

**Background:**

HIV clinical presentation in the acute stage is variable and some of its virological and immunological aspects are not completely understood. Most cases of HIV- associated reactive hemophagocytic syndrome have been reported in patients with advanced stages of HIV and to our knowledge, there are only 8 cases in the English literature presenting during acute HIV infection, most in East Asia, being this the first case in a European patient.

**Case presentation:**

We report a case of a European Caucasian 27- year old woman with a primary HIV- infection presenting with extremely low CD4+ T cell count who developed a haemophagocytic syndrome after starting ART and in whom we documented a very unusual serological and virological response, characterized by an impaired HIV- antibody production and a 12 month time frame to reach an undetectable viral load, despite no evidence of resistance.

**Conclusions:**

This case report apart from describing an unusual clinical presentation of an acute HIV infection as hemophagocytic syndrome provides useful information that might contribute for understanding some subtle issues in acute HIV infection, namely the dynamics of virological and immunological aspects after antiretroviral therapy initiation.

**Electronic supplementary material:**

The online version of this article (doi:10.1186/s12879-016-1945-9) contains supplementary material, which is available to authorized users.

## Background

Acute Human Immunodeficiency Virus (HIV) infection clinical presentation is variable and some of its virological and immunological aspects are not completely understood.

We report a case of a woman with a primary HIV- infection who developed a hemophagocytic syndrome and in whom we documented a very unusual serological and virological response, characterized by an impaired HIV- antibody production and a 12 month time frame to reach an undetectable viral load.

## Case presentation

A 27 year old female, presented to our hospital on 5^th^ July 2013 with fever, throat pain, non productive cough, anorexia and weight loss (4 kg) for one month duration.

The patient recalled unprotected sexual intercourse with a steady partner 2 months before symptoms presentation. There was no history of intravenous drug use, blood transfusions or past surgical procedures. She had performed a mandatory HIV test before her departure from Eastern Europe, in August 2012, which was negative.

On admission the patient vital signs were: temperature of 39, 5 °C, blood pressure 110/65 mmHg, pulse rate 102 beats per minute and respiratory rate 16 breaths per minute.

White exudates were evident in oropharyngeal examination. Neither rash nor palpable cervical, axillary or inguinal lymph nodes were present.

Routine laboratory revealed pancytopenia: Hemoglobin (Hb) was 7, 9 g/dL, White blood cells (WBC) was 1.72 × 10^9^/L with 0.79 × 10^9^/L neutrophils, 0.40 × 10^9^/L lymphocytes and 88 × 10^9^/L platelets.

C-reactive protein (CRP) was 0, 6 mg/L (normal range < 3 mg/L). Renal function and liver tests were normal.

HIV screening was positive and the immunoblot assay- INNO-LIA™ indeterminate. An additional file describes the tests in more detail [See Additional file 1].

**Fig. 1:**
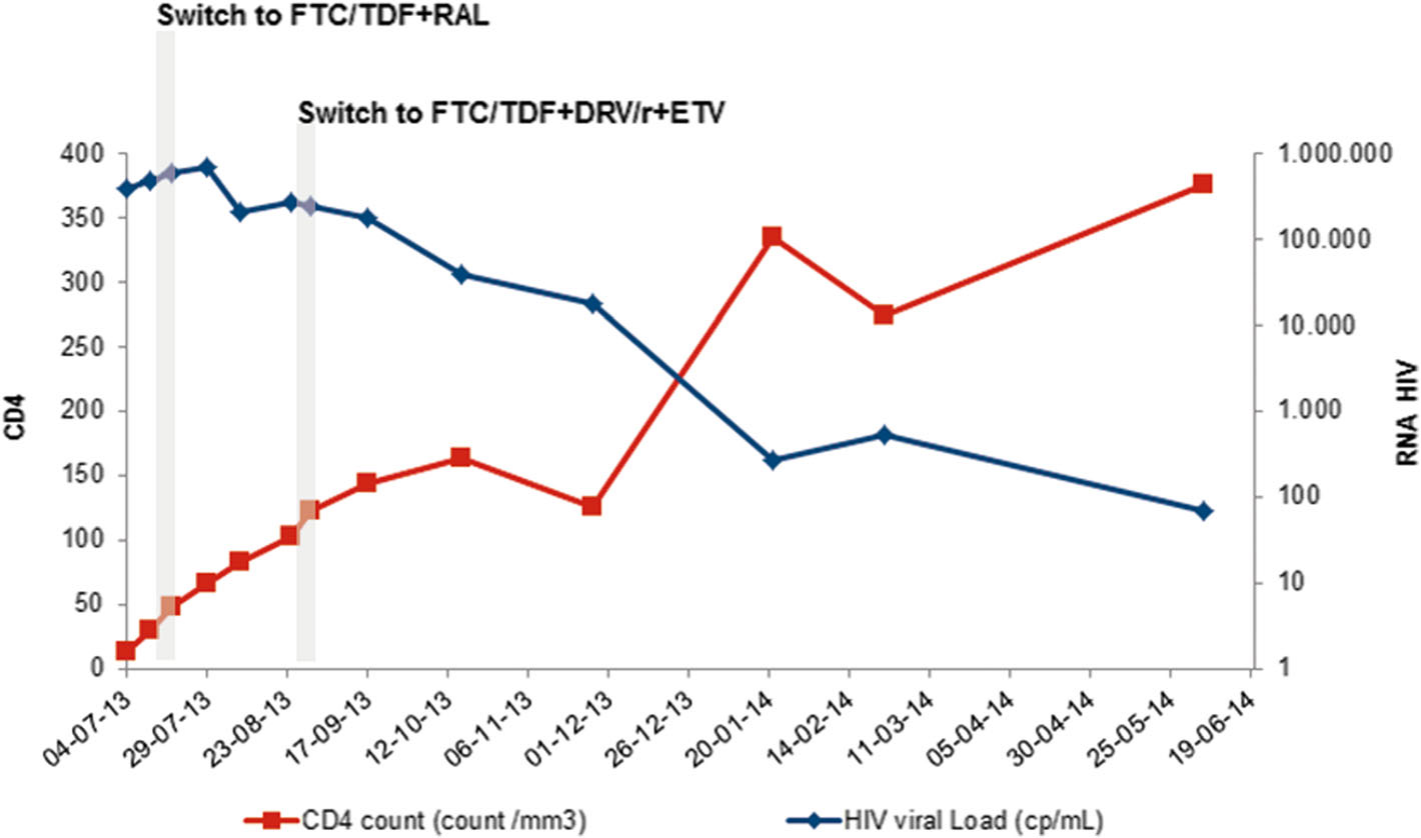
CD4 count and HIV viral load dynamics over 12 months after HIV diagnosis and ART initiation

She was admitted to the Infectious Diseases Department with the presumed diagnosis of an acute HIV-infection. RNA viral load in plasma was of 384.687 cp/mL (5, 59 log 10), CD4+ cell count was 13/mm3. A type 1, subtype A HIV virus was identified (Fig. 1).

At admission: blood cultures were sterile; Monospot test negative. Serological studies revealed: IgG positive with IgM negative for Epstein barr Virus (EBV), *Toxoplasma gondii*, Cytomegalovirus (CMV) and *Herpes simplex 1*; IgG and IgM were negative for *Herpes simplex* 2. *Treponema pallidum* particle agglutination assay, cryptococcal antigen, CMV antigen and CMV viral load in blood also negative.

Chest X-Ray, abdominal ultrasound, chest and abdominal CT scan did not reveal relevant abnormality.

Antiretroviral therapy (ART) was started on 11th July with tenofovir/emtricitabine and atazanavir/ritonavir.

The patient maintained fever but had no focal symptoms.

Asymptomatic progressive liver enzymes elevation was documented (aspartate aminotransferase/alanine aminotransferase (AST/ALT): 153/80UI/L; alkaline phosphatase (ALK) 321UI/L, total bilirubin 3.89 mg/dL).

The results of genotypic resistance test became available and revealed no significant mutations that could confer resistance either to protease or reverse transcriptase inhibitors.

**Fig. 2:**
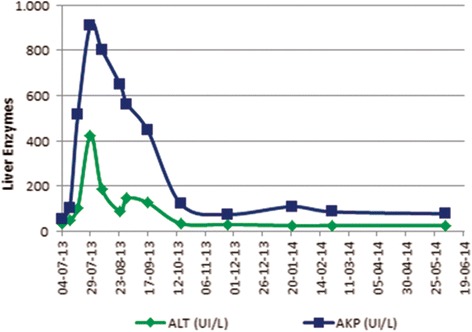
Liver enzymes evolution

At 7^th^ day ritonavir was/ritonavir was switched to raltegravir (Fig. 1). Despite this, liver cytolysis/cholestasis continued worsening (Fig. 2) and was accompanied by aggravated pancytopenia.

Lactate dehydrogenase (LDH) and Beta-2 microglobulin were elevated: 1872 mg/dL (Normal <225 mg/dL) and 4750 mg/dL (Normal < 2530 mg/dL), respectively.

Ferritin was of 2095 ng/ml (Normal: 10–120 ng/mL), triglycerides was of 254 mg/dL (Normal < 150 mg/dL). Fibrinogen was diminished (1, 14 g/L), as NK activity (0, 09 %).

**Fig. 3:**
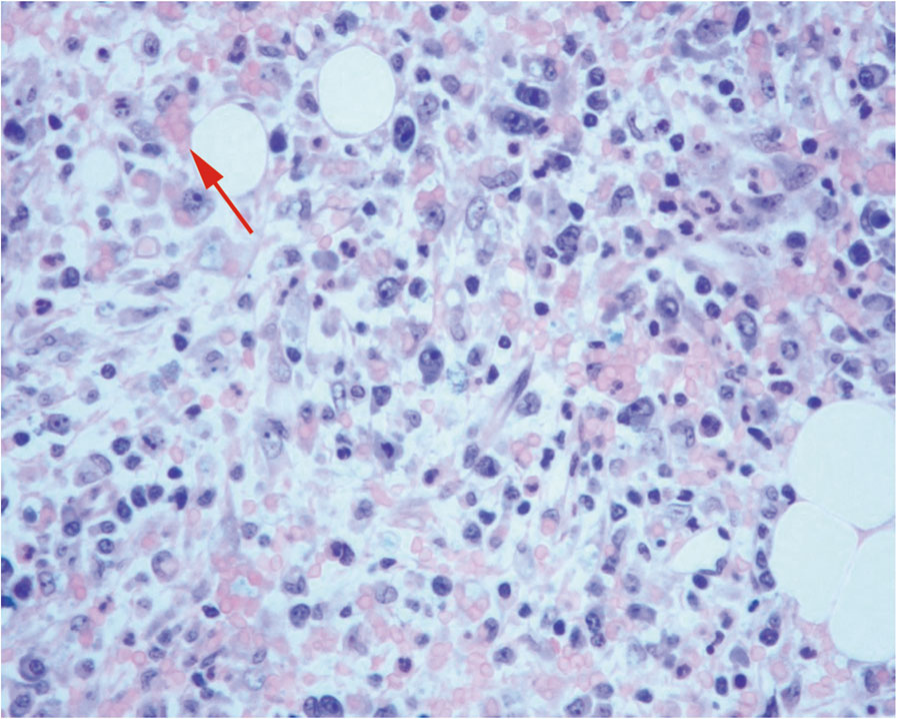
Bone Marrow Biopsy: activated macrophages engulfing erythrocytes suggesting HS

Bone marrow histological examination (18^th^ July) revealed: hypercelularity and architectural disorganization, erythroid hyperplasia with dyserythropoiesis, lymphoid aggregates, perivascular and intersticial plasmocytosis and activated macrophages engulfing erythrocytes. No evidence of mycobacteria or CMV infection was present (both excluded by cultural exam and polymerase chain reaction (PCR)) (Fig. 3).

Liver biopsy (25^th^ July) revealed: portal inflammatory infiltrates, disperse necroinflammatory lesions, moderate cholestasis, hepatocyte ballooning and multifocal esteatosis.

At this stage, a diagnosis of Hemophagocytic Syndrome was assumed, according to the Haemophagocytic Histiolymphocytosis (HLH) -2004 criteria. Repeated blood cultures (including for mycobacterium), cryptococcal antigen, CMV PCR and antigen and EBV PCR were negative. CRP was within normal range along this period. ART was maintained and steroids were started (prednisolone 80 mg/day).

At four weeks of therapy, viral load had not declined significantly (Fig. 1). At this point, genotypic drug resistance test was repeated and again no relevant mutations were identified.

At 5 weeks of therapy, fever and asthenia persisted with no other symptoms.

At 6th week viral load increased to 268.211cp/mL (Fig. 1). Esophageal candidiasis and cytomegalic reactivation without organ involvement were documented and treated.

A group decision was to optimize ART to tenofovir/emtricitabine + darunavir/ ritonavir + etravirine (TDF/FTC + DRV/r + ETV) (Fig. 1). The patient was on Daily Observed Therapy.

A third genotypic resistance test was performed and again no relevant or new mutations were found, using different interpretation algorithms: Stanford HIVdb and HIV REGA algorithms (version 8.0.2; available at http://www.rega.kuleuven.be/cev/). An additional file describes this in more detail [See Additional file 2].

INNO-LIA™ HIV I/II test was repeated and an indeterminate result was obtained once again with the same reactivity rating for gp41.

Patient’s condition progressively improved, apyrexia was documented and she was discharged, with a progressively reducing doses of steroids, by 20^th^ of September (after ~11 weeks of hospital stay; ~ 9 weeks of FTC/TDF + ETV + DRV/r).

Subsequent viral load quantifications revealed no significant decline (Fig. 1).

Given this unexpected response to therapy a blood sample was sent to Monogram Biosciences® (San Francisco, USA), for phenotypic and genotypic resistance testing: the virus was sensitive to all Nucleoside and Non-Nucleoside Reverse Transcriptase Inhibitors (NRTIs/NNRTIs) and PI drugs. An additional file describes this in more detail [See Additional file 3].

Therapeutic drug monitoring (TDM) was also performed and TDF/FTC + DRV/r + ETV were in therapeutic levels.

Three plasma samples from different dates (August, September and November 2013) were sent to INNOGENETICS reference laboratory in Belgium for HIV testing confirming the INNO-LIA HIV Score results of our institution. Based on those results, they concluded that this patient was not developing expected antibody reactivity. An additional file describes this in more detail [See Additional file 4].

Determination of specific antibodies against Pneumococcal capsule polysaccharides and tetanus toxoid were also performed and results were below reference values.

Hepatitis B surface antibody (HBsAb), after 2 doses of hepatitis B vaccine, was non quantifiable.

Almost a year after HIV diagnosis and ART initiation, HIV viral load was of 70cp/mL (1, 85 Log10) and CD4+ count of 376/mm3 (04/06/2014). ART was maintained (Fig. 1).

In July 2014, the patient returned to her country and given the unavailability of the current regimen in her homeland, ART was switched to tenofovir/emtricitabine + efavirenz. Patient remained asymptomatic and in the last determination dating of 14^th^ July 2014 HIV blood viral load was not detectable (<20cp/mL as threshold) and CD4+ cell count was of 312/mm3.

## Conclusions

We report a case of an acute HIV infection with a rare and intriguing clinical, virological and serological behaviour.

Considering the potential benefits of early treatment [1–3], ART was started, with a PI based regimen while waiting for genotypic resistance test results. Despite this, patient condition got worse and clinical, analytical and histological findings were consistent with the diagnosis of hemophagocytic syndrome [4, 5]. This is itself a rare condition during acute HIV infection [6–8].

Whether hemophagocytic syndrome in this case is attributable to HIV itself, to ART, to an immune reconstitution syndrome (IRIS) or other cause, is a matter of interesting debate.

In our patient hemophagocytic syndrome was diagnosed early after ART introduction, persisted after changing the drug regimen and despite the absence of virological and immune response to therapy, all facts that argue against ART or IRIS as causes. Other possible precipitating factors such as latent virus reactivation were also excluded at that stage. For us the most likely explanation is that hemophagocytic syndrome was a manifestation of the complex inflammatory storm occurring in acute HIV infection. Although we have started steroids (80 mg/daily) soon after the diagnosis, the response to this therapy was slow. As in other secondary Hemophagocytic syndromes, anti-cytokine and anti-proliferative therapy (such etoposide, cyclosporine, etc.) might have been of benefit but given the clinical stability and the possible deleterious effects of more imunosupression we were not favourable to the use of these options.

Apart from the aetiology of the hemophagocytic syndrome, the strange dynamic of HIV viral load after ART initiation and the persistence of an “indeterminate” INNO-LIA HIV testing are all worth of discussion.

Even without treatment, viral load usually declines over 2–6 months in acute HIV infection, concurrent with the development of HIV-specific immune responses, to approach a steady state in which viral production and clearance are relatively matched [9].

It is difficult to explain why the viral load firstly increased on therapy and then had an extremely slow decline, having all plausible causes excluded, namely genotypic viral resistance, phenotypic viral resistance or infra therapeutic drugs levels. It is possible that this atypical virological kinetic occurring despite adequate anti-viral therapy was a result of unchecked macrophage/monocyte activation, resulting in rapid/unchecked HIV virus turnover from these activated cells.

Concerning the peculiar serological findings some could hypothesize that ART initiation early at diagnosis of acute HIV infection might have contributed for the persistence of an “indeterminate” INNO-LIA HIV testing. According to David Montefiori et al., early ART has a suppressive effect on the normal antibody response to HIV-1, presumably by limiting the concentration of viral antigens needed to drive virus-specific B-cell maturation [10, 11].

In our patient however, ART initiation had no significant impact on the concentration of virus at an early stage, meaning that the inhibition of immunological response due to the suppressive effect of ART is not plausible in this case.

For us, the abnormal antibody response can be linked to the unchecked and disordered B-cell help and activation that may occur as part of the hemophagocytic phenomenon. Also, we believe that the patients’ immune system has somewhat contributed for this unusual picture. In acute HIV infection, viral decline is concurrent with the development of specific HIV antibodies. In our case, even after achieving an almost “undetectable viral load” and after hemophagocytic syndrome resolution, HIV confirmatory test was repeatedly “indeterminate” concluding that the patient did not produce some of the specific antibodies against HIV.

This hypothesis is further supported by abnormal levels of specific antibodies against Pneumococcal capsule polysaccharides and tetanus toxoid. It is possible that the acquired immunodeficiency attributable to HIV has supervened to an innate disturbance on the patient’s immune system, which we were unable to identify.

Acute HIV infection treatment decision is made on extrapolation from what is defined for chronic HIV infection, as clinical trials are scarce.

Case reports like ours might contribute for understanding some subtle issues in acute HIV infection, namely the dynamics of virological and immunological aspects.

## Additional files

Additional file 1: HIV screening was performed with ARCHITECT HIV Ag/Ab Combo assay™ (ABBOTT®) for the simultaneous qualitative detection of HIV-1 p24 antigen and antibodies to HIV-1 (HIV-1 group M and group O) and HIV-2 and a reactive result (S/CO = 146.5) was obtained. The immunoblot assay- INNO-LIA™ HIV I/II Score (Innogenetics®) was further performed in the same plasma sample. LiRAS™ (Line Reader and Analysis Software) was used for automated interpretation and an indeterminate result was readable (gp41: 3+ and p31: 1+). (DOC 26 kb)
Additional file 2: Genotypic resistance test (ViroSeq™ HIV-1 Genotyping System v2.0™; ABI Prism 3100 Genetic Analyser™; ViroSeq HIV-1™ Genotyping System Software v2.8; ABBOTT®) and the results interpreted by the Stanford University HIV Drug Resistance Database (Stanford HIV db, version 6.0.11, available at http://hivdb.stanford.edu/). (DOC 26 kb)
Additional file 3: Phenotypic resistance testing (PhenoSense GT ®): The results indicated that the virus (HIV-1 subtype A) was sensitive to all NRTI/NNRTI and PI drugs, as by the phenotypic as genotypic interpretation (list of mutations detected: RT – V35T, T39R, E40D, K49R, V80I, Q102K, K122E, D123S, I42M, A158S, C182S, K173S, Q164K, Q197L, T200A, Q207A, R211S, E248D, A272P, R277K, T286A, E291D, V292I, P294T, L295M; PR – E35D, M36I, R41K, I62I/v, H69K, T74T/S, L89M). The combination phenotype/genotype net assessment showed that the virus was sensitive to all drugs with a virus replication capacity of 88 %. (DOC 26 kb)
Additional file 4: Three plasma samples from different dates (August, September and November 2013) were sent to INNOGENETICS reference laboratory in Belgium for HIV testing (HIV-1 p24 antigen and immunoblotting). The results were: only one sample (August 2013) gave a reactive result for the presence of the p24 antigen; all 3 samples gave an identical result on INNO-LIA HIV Score - indeterminate (with gp41 3+; p31 +/− or -); confirming the INNO-LIA HIV Score results of our institution. Based on the INNO-LIA HIV Score results, they concluded that this patient was not developing common or expected antibody reactivity. (DOC 26 kb)

## Abbreviations

ALK: Alkaline phosphatise
ALT: Alanine aminotransferase
ART: Antiretroviral therapy
AST: Aspartate aminotransferase
CMV: Cytomegalovirus
CRP: C-reactive protein
DRV: Darunavir
EBV: Epstein barr virus
ETV: Etravirine
FTC: Emtricitabine
Hb: Hemoglobin
HBs Ab: Hepatitis B surface antibody
HIV: Human immunodeficiency virus
IRIS: Immune reconstitution syndrome
LDH: Lactate dehydrogenase
NNRTIs: Non-nucleoside reverse transcriptase inhibitors
NRTIs: Nucleoside reverse transcriptase inhibitors
PCR: Polymerase chain reaction
PI: Protease inhibitor
R: Ritonavir
TDF: Tenofovir
TDM: Therapeutic drug monitoring
WBC: White blood cells

## References

[1] O’Brien M, Markowitz M (2012). Should we treat acute HIV infection?. Curr HIV/AIDS Rep.

[2] Bell SK, Little SJ, Rosenberg ES (2010). Clinical management of acute HIV infection: best practice remains unknown. J Infect Dis.

[3] AIDS info 2015. Guidelines for the Prevention and Treatment of Opportunistic Infections in HIV-Infected Adults and Adolescents. Available at: https://aidsinfo.nih.gov/guidelines/html/4/adult-and-adolescent-oi-prevention-and-treatment-guidelines/325/tb. Accessed 7 May 2013.

[4] Henter JI, Horne A, Arico M, Egeler RM, Filipovich AH, Imashuku S (2007). HLH-2004: diagnostic and therapeutic guidelines for hemophagocytic lymphohistiocytosis. Pediatr Blood Cancer.

[5] Antonio C, Todaro G, Bonina L, IAria C (2011). Letter to the editor: please, do not forget secondary hemophagocytic lymphohistiocytosis in HIV- infected patients. Int J Infect Dis.

[6] Sailler L, Duchayne E, Marchou B (1997). Etiological aspects of reactive hemophagocytoses: retrospective study in 99 patients. Rev Med Interne.

[7] Fardet L, Lambotte O, Meynard JL (2010). Reactive haemophagocytic syndrome in 58 HIV-1 infected patients: clinical features, underlying diseases and prognosis. AIDS.

[8] Adachi E, Koibuchi T, Imai K (2013). Hemophagocytic syndrome in an acute human immunodeficiency virus infection. Intern Med.

[9] Michael KJ, Youn HL, Hazelwood JD (2008). Treatment response in acute/early infection versus advanced AIDS: equivalent first and second phases of HIV RNA decline. AIDS.

[10] Montefiori D, Hill TS, VO HTT, Walker BD, Rosenberg ES (2001). Neutralizing antibodies associated with viremia control in a subset of individuals after treatment of Acute Human Immunodeficiency Virus Type 1 Infection. J Virol.

[11] Altfeld M, Rosenberg ES, Shankarappa R (2001). Cellular immune responses and viral diversity in individuals treated during acute and early HIV-1 infection. J Exp Med.

