# Supplementary material for: Acute HIV infection presenting as hemophagocytic syndrome with an unusual serological and virological response to ART
Source: BMC Infect Dis. 2016 Oct 28;16:619. doi: 10.1186/s12879-016-1945-9 (PMC5086040; doi:10.1186/s12879-016-1945-9)
Supplement: Additional file 1: — HIV screening was performed with ARCHITECT HIV Ag/Ab Combo assay™ (ABBOTT®) for the simultaneous qualitative detection of HIV-1 p24 antigen and antibodies to HIV-1 (HIV-1 group M and group O) and HIV-2 and a reactive result (S/CO = 146.5) was obtained. The immunoblot assay- INNO-LIA™ HIV I/II Score (Innogenetics®) was further performed in the same plasma sample. LiRAS™ (Line Reader and Analysis Software) was used for automated interpretation and an indeterminate result was readable (gp41: 3+ and p31: 1+). (DOC 26 kb) [file 12879_2016_1945_MOESM1_ESM.doc]

Additional File 1- HIV screening was performed with **ARCHITECT** HIV Ag/Ab Combo assay™ (ABBOTT®) for the simultaneous qualitative detection of HIV-1 p24 antigen and antibodies to HIV-1 (HIV-1 group M and group O) and HIV-2 and a reactive result (S/CO = 146.5) was obtained. The immunoblot assay- INNO-LIA™ HIV I/II Score (Innogenetics®) was further performed in the same plasma sample. LiRAS™ (Line Reader and Analysis Software) was used for automated interpretation and an indeterminate result was readable (gp41: 3+ and p31: 1+).
