# Supplementary material for: Acute HIV infection presenting as hemophagocytic syndrome with an unusual serological and virological response to ART
Source: BMC Infect Dis. 2016 Oct 28;16:619. doi: 10.1186/s12879-016-1945-9 (PMC5086040; doi:10.1186/s12879-016-1945-9)
Supplement: Additional file 2: — Genotypic resistance test (ViroSeq™ HIV-1 Genotyping System v2.0™; ABI Prism 3100 Genetic Analyser™; ViroSeq HIV-1™ Genotyping System Software v2.8; ABBOTT®) and the results interpreted by the Stanford University HIV Drug Resistance Database (Stanford HIV db, version 6.0.11, available at http://hivdb.stanford.edu/). (DOC 26 kb) [file 12879_2016_1945_MOESM2_ESM.doc]

Additional File 2- Genotypic resistance test (ViroSeq™ HIV-1 Genotyping System v2.0™; ABI Prism 3100 Genetic Analyser™; ViroSeq HIV-1™ Genotyping System Software v2.8; ABBOTT®) and the results interpreted by the Stanford University HIV Drug Resistance Database (Stanford HIV db, version 6.0.11, available at <http://hivdb.stanford.edu/>)
