# Supplementary material for: Acute HIV infection presenting as hemophagocytic syndrome with an unusual serological and virological response to ART
Source: BMC Infect Dis. 2016 Oct 28;16:619. doi: 10.1186/s12879-016-1945-9 (PMC5086040; doi:10.1186/s12879-016-1945-9)
Supplement: Additional file 3: — Phenotypic resistance testing (PhenoSense GT ®): The results indicated that the virus (HIV-1 subtype A) was sensitive to all NRTI/NNRTI and PI drugs, as by the phenotypic as genotypic interpretation (list of mutations detected: RT – V35T, T39R, E40D, K49R, V80I, Q102K, K122E, D123S, I42M, A158S, C182S, K173S, Q164K, Q197L, T200A, Q207A, R211S, E248D, A272P, R277K, T286A, E291D, V292I, P294T, L295M; PR – E35D, M36I, R41K, I62I/v, H69K, T74T/S, L89M). The combination phenotype/genotype net assessment showed that the virus was sensitive to all drugs with a virus replication capacity of 88 %. (DOC 26 kb) [file 12879_2016_1945_MOESM3_ESM.doc]

Additional File 3- Phenotypic resistance testing (PhenoSense GT ®): The results indicated that the virus (HIV-1 subtype A) was sensitive to all NRTI/NNRTI and PI drugs, as by the phenotypic as genotypic interpretation (list of mutations detected: RT – V35T, T39R, E40D, K49R, V80I, Q102K, K122E, D123S, I42M, A158S, C182S, K173S, Q164K, Q197L, T200A, Q207A, R211S, E248D, A272P, R277K, T286A, E291D, V292I, P294T, L295M; PR – E35D, M36I, R41K, I62I/v, H69K, T74T/S, L89M). The combination phenotype/genotype net assessment showed that the virus was sensitive to all drugs with a virus replication capacity of 88%.
