# Supplementary material for: Acute HIV infection presenting as hemophagocytic syndrome with an unusual serological and virological response to ART
Source: BMC Infect Dis. 2016 Oct 28;16:619. doi: 10.1186/s12879-016-1945-9 (PMC5086040; doi:10.1186/s12879-016-1945-9)
Supplement: Additional file 4: — Three plasma samples from different dates (August, September and November 2013) were sent to INNOGENETICS reference laboratory in Belgium for HIV testing (HIV-1 p24 antigen and immunoblotting). The results were: only one sample (August 2013) gave a reactive result for the presence of the p24 antigen; all 3 samples gave an identical result on INNO-LIA HIV Score - indeterminate (with gp41 3+; p31 +/− or -); confirming the INNO-LIA HIV Score results of our institution. Based on the INNO-LIA HIV Score results, they concluded that this patient was not developing common or expected antibody reactivity. (DOC 26 kb) [file 12879_2016_1945_MOESM4_ESM.doc]

Additional File 4- Three plasma samples from different dates (August, September and November 2013) were sent to INNOGENETICS reference laboratory in Belgium for HIV testing (HIV-1 p24 antigen and immunoblotting). The results were: only one sample (August 2013) gave a reactive result for the presence of the p24 antigen; all 3 samples gave an identical result on INNO-LIA HIV Score - indeterminate (with gp41 3+; p31 +/- or -); confirming the INNO-LIA HIV Score results of our institution. Based on the INNO-LIA HIV Score results, they concluded that this patient was not developing common or expected antibody reactivity.
